# Applying the FAIR principles to data in a hospital: challenges and opportunities in a pandemic

**DOI:** 10.1186/s13326-022-00263-7

**Published:** 2022-04-25

**Authors:** Núria Queralt-Rosinach, Rajaram Kaliyaperumal, César H. Bernabé, Qinqin Long, Simone A. Joosten, Henk Jan van der Wijk, Erik L.A. Flikkenschild, Kees Burger, Annika Jacobsen, Barend Mons, Marco Roos, BEAT-COVID Group, COVID-19 LUMC Group

**Affiliations:** 1 Department of Human Genetics, Leiden University Medical Center, Leiden, The Netherlands; 2 Department of Infectious Diseases, Leiden University Medical Center, Leiden, The Netherlands; 3 Department of Biomedical Data Sciences, Leiden University Medical Center, Leiden, The Netherlands; 4 Department of IT&DI, Leiden University Medical Center, Leiden, The Netherlands; 5 GO FAIR Foundation, Leiden, The Netherlands; 6 CODATA, Paris, France

**Keywords:** Patient data, Ontologies, FAIR, Research data management, Hospital, Open science

## Abstract

**Background:**

The COVID-19 pandemic has challenged healthcare systems and research worldwide. Data is collected all over the world and needs to be integrated and made available to other researchers quickly. However, the various heterogeneous information systems that are used in hospitals can result in fragmentation of health data over multiple data ‘silos’ that are not interoperable for analysis. Consequently, clinical observations in hospitalised patients are not prepared to be reused efficiently and timely. There is a need to adapt the research data management in hospitals to make COVID-19 observational patient data machine actionable, i.e. more Findable, Accessible, Interoperable and Reusable (FAIR) for humans and machines. We therefore applied the FAIR principles in the hospital to make patient data more FAIR.

**Results:**

In this paper, we present our FAIR approach to transform COVID-19 observational patient data collected in the hospital into machine actionable digital objects to answer medical doctors’ research questions. With this objective, we conducted a coordinated FAIRification among stakeholders based on ontological models for data and metadata, and a FAIR based architecture that complements the existing data management. We applied FAIR Data Points for metadata exposure, turning investigational parameters into a FAIR dataset. We demonstrated that this dataset is machine actionable by means of three different computational activities: federated query of patient data along open existing knowledge sources across the world through the Semantic Web, implementing Web APIs for data query interoperability, and building applications on top of these FAIR patient data for FAIR data analytics in the hospital.

**Conclusions:**

Our work demonstrates that a FAIR research data management plan based on ontological models for data and metadata, open Science, Semantic Web technologies, and FAIR Data Points is providing data infrastructure in the hospital for machine actionable FAIR Digital Objects. This FAIR data is prepared to be reused for federated analysis, linkable to other FAIR data such as Linked Open Data, and reusable to develop software applications on top of them for hypothesis generation and knowledge discovery.

## Background

The COVID-19 pandemic has challenged healthcare and research data management systems worldwide to provide reusable patient data for rapid and efficient translational research. Clinical data, laboratory measurements, and various omics data such as transcriptomics and metabolomics, are routinely collected from hospitalized COVID-19 patients to inform medical doctors about patients’ health status and to support research on treatment options. Analysing data integrated from multiple sources in a hospital, complemented with data from other hospitals and public knowledge bases, can generate critical information about disease mechanisms to support diagnosis, prognosis and decisions on interventions. However, research and clinical data are often not prepared for instant secondary use involving multiple sources. This was already an obstacle for efficient clinical and biomedical research in general, but a pandemic of a poorly understood novel disease that overloads hospitals’ capacity has revealed the significance of this problem.

Integrative analysis is challenged by software systems used to collect these various types of data from patients in hospitals. Different formats may be used (e.g. CSV or JSON) and the semantics of data are often underspecified and captured in a proprietary syntax or by different standards (e.g. HL7 FHIR or OpenEHR). This can result in fragmentation over multiple ‘silos’ that are not sufficiently interoperable for instant computational analysis. Reuse and reproducibility are further hampered by missing or unstandardised provenance, such as the time and date at which data were collected (e.g. scans may be performed on a different day than blood measurements). Furthermore, to expand analysis beyond one hospital, information on consent and regulations that control data access, reuse, and sharing are often unclear and not easily assessable. Complete harmonization of access regulations between institutes and countries is not realistic, but analysis could still be efficient if access regulations were at least computationally assessable.

Ideally, hospital systems are set up with integrative, federated data analytics in mind. Global leaders in data science have posed that this can be achieved by applying agreed upon standards to make data globally findable, accessible, interoperable, and reusable for humans and computers, also referred to by as ‘the FAIR principles’ [1]. Indeed, projects such as the GO FAIR Virus Outbreak Data Network (VODAN) [2], the ZonMW Covid program [3], the Trusted World of Corona (TWOC) [4], and ELIXIR Covid project [5] embrace FAIR principles as a key element of their COVID-19 data management strategy. A quintessential objective is turning data and data containers into machine actionable FAIR Digital Objects (FDOs), in this paper defined as resources in a digital, machine understandable form including explanatory metadata and addressable by a globally unique persistent and resolvable identifier; a formal framework for FDOs is under development, see [6, 7]. This will optimize the ability to integrate and visualise data from many sources, facilitate fine-grained data access regulation, and allow for decentralised and machine assisted analysis [8]. The latter is further enabled by the development of infrastructure that supports ‘data visiting’ [9, 10]. This is attractive for clinical data because (i) existing systems can be complemented with data visiting functions, thereby keeping their other functions in place, (ii) the output of an analysis is generally less privacy sensitive than the input. In Europe, the General Data Protection Regulation (GDPR) policy supports data visiting by requiring that access regulations for personal data are clearly defined [11].

Methods to facilitate the implementation of FAIR principles, or ‘FAIRification’, are currently being investigated in multiple projects and initiatives. We use ‘FAIRification’ to denote the *process* towards achieving FAIRification goals, irrespective of specific implementation choices per principle. We have previously published a generic workflow [12], as a basis for specialised variations such as for rare disease registries [13]. Related activities are the development of the FAIR cookbook in the FAIRplus project [14, 15], the three point framework for FAIRification of metadata by the VODAN GO FAIR network [16], and the organisation of a FAIRification steward team to support rare disease registries reach their FAIR goals [17]. The application of FAIR principles in hospitals is starting to be adopted in Europe as a key strategy for nationwide healthcare research data infrastructure [18, 19]. Cross connections through multinational collaborations, such as in ELIXIR and GO FAIR, and domain specific collaborations such as via globally operating patient organisations, could support convergence of FAIR implementation choices to further facilitate the adoption of FAIR principles and thereby efficient analysis across multiple hospitals in multiple countries.

At the Leiden University Medical Centre (LUMC), the implementation of FAIR principles for COVID-19 data is part of a multidisciplinary collaboration, coined ‘The BEAT-COVID project’. This collaboration was initiated in March 2020 to face the multiple analysis challenges of the COVID-19 pandemic. The LUMC is a tertiary care, teaching and research hospital in the Netherlands that encompasses clinical and research groups with expertise on immunology, biomedicine, data management and data science. The groups work together on collecting and sharing different types of patient data, analyses, findings, expertise, and novel solutions implemented in the hospital (e.g. see [20]). One of the challenges is to implement a FAIR Research Data Management plan (RDM) comprising FAIRification of priority resources and a FAIR based architecture that complements the existing data management systems in the hospital.

**Fig. 1:**
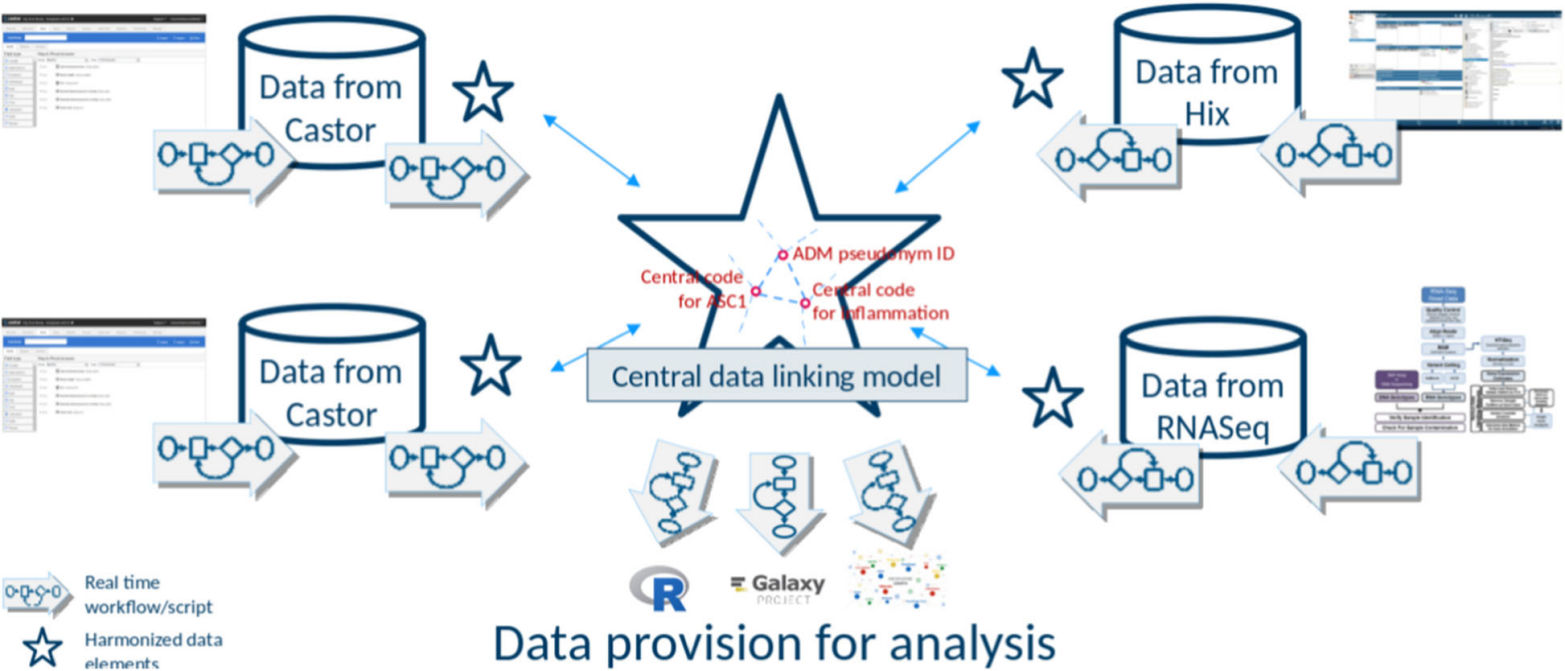
Illustration of the central concepts of the envisioned FAIR based architecture: the central star represents the data linking model for interoperability that the sources refer to (data and metadata), the small stars next to each source represent what is used of the central model to describe the source (thereby becoming ‘self-describing’), the arrows represent workflows or scripts: for the source systems to map or convert source data and metadata to the central data linking model, for retrieving data from across the sources through the central data linking model, and for analysis. FAIR Data Points provide access to the ‘ontologised’ metadata and data (not shown)

We hypothesise that the use of existing ontologies and ontological models will enable turning patient data into machine readable digital objects that are prepared for secondary use. Our objective is to develop ontological models that represent and link the data records and metadata of the datasets in the existing LUMC data management systems (Fig. 1). In our ontology centred approach, data can stay in existing systems but are made accessible ’in terms of’ the central data linking model to create a virtual warehouse. We reused existing ontological models such as the core ontological model for common data elements developed in the European Joint Programme on Rare Diseases (EJP RD) for patient registries [21], and the Data Catalogue Vocabulary (DCAT) for datasets [22]. The metadata is made accessible by a FAIR Data Point (FDP) instance [23]. FDPs ensure that BEAT-COVID resources can be found and used through querying machine readable metadata. It includes the pointers to access the content of the resource for analysis workflows, if access is permitted. By using ontologies, patient data in the hospital are virtually linked with other ontologically described data in the hospital, but also public Linked ‘Open’ Data (LOD). This can boost the potential for knowledge discovery and data+knowledge driven analytics. Interestingly, ontologies may also be used to describe data access restrictions [24, 25] to complement FAIR metadata with information that supports data safety and patient privacy.

In this paper, we describe and implement our approach for FAIRification of COVID-19 observational patient data in an academic hospital. We selected cytokine measurements of hospitalised patients as our primary objective of FAIRification and development of the FAIR RDM. We synthesized an artificial dataset mimicking original laboratory data obtained from patient samples to study the data lifecycle without the risk of violating patient privacy. Our main result is the FAIRification in the hospital. We also show that our FAIRifcation approach is providing cytokine measurements as FDOs and is enabling applications on top of this FAIR patient data for analytics. Importantly, this work has been done in close collaboration with clinicians and data managers who are familiar with the existing hospital data systems and data lifecycles to establish best practices for making data FAIR in the hospital. We demonstrate that a FAIR RDM plan based on describing data and metadata by ontologies delivers an infrastructure that complements existing infrastructure with FDOs that are prepared for integrative and federated analysis. We show our first results and the solutions that are currently being developed as LUMC research data management procedures. We finally discuss what FAIRification entails in a ‘real world’ hospital situation involving different stakeholders and departments, and future challenges such as data access regulation in a FAIR ecosystem.

## Results

### FAIR status of patient data in existing systems

Our FAIR assessment of the cytokine data in existing systems revealed that while the structure and findability improved with each step of the data management lifecycle, no FAIR standards were applied to make the data and metadata globally understandable ‘for machines’, such as for automated computer processing (Table 1). The original data from the clinical laboratory that measured the cytokine levels was well structured, but not in a uniform, globally machine readable way. The data were further pre-processed manually and transferred to the Electronic Data Capture (EDC) software Castor [26]. Although this captured data electronically and in a uniform way, there were no ontological representations added to the data collection forms to create a FAIR dataset. Data was subsequently transferred from Castor into the Opal data warehouse system [27], conform the standard workflow for preparing data for research at the LUMC. Opal is a generic system to bring datasets from different systems in the hospital into one warehouse, supporting transformations and annotation on the data level with a vocabulary chosen by the user. Opal provides researchers at the LUMC a central access point to research data that are syntactically machine readable. It offers APIs that bioinformaticians can incorporate in their workflows. Anonymised data of daily parameters from patient records was imported into Opal without including retrievable patient identifiers in the research environment of the hospital on almost real time.

**Table 1:** FAIR assessment of existing systems containing cytokine data

Existing system	FAIR assessment
Original cytokine dataset (Excel)	Structured, but custom-built, thus not in a uniform, globally machine readable way.
Dataset in Castor EDC	Structured in a uniform way, but no standards were applied to create a FAIR dataset.
Dataset in Opal	Structured, findable through the central LUMC warehouse and accessible through an API, but no global machine readable standards were applied to represent the data and metadata for machine processing.

Opal’s native metadata tool Mica [28] provides annotation on the dataset level such as how, when, where, by whom, under what conditions data has been collected. This information is subsequently published in a Web portal. Therefore, Mica provides resource information that is human readable on the Web. This metadata is not also available in a machine readable form. Findability for machines can be improved by adding a machine readable ontological representation. Our automated FAIR assessment of a dataset described in Mica (see here) showed specifically which FAIR improvements could be made to make the metadata descriptions in Mica more machine actionable and standardized. Although Mica implements unique identifiers, these were not persistent in our case, and they were also not explicitly defined in the metadata. This creates challenges for data accessibility and reusability. Some systems, notably Opal [29], provide handles to integrate FAIR features, but we chose to first incorporate independent components to minimize requirements for other systems and thereby optimize reusability of the approach.

### Coordinated FAIRification

A coordinated FAIRification process with BEAT-COVID colleagues was set up to improve the machine readability, global interoperability, and findability of the COVID-19 data. We developed ontological models for data record in collaboration with data collectors, data managers, data analysts and medical doctors. Similarly, we developed machine actionable metadata to improve the findability, accessibility, and reusability of the datasets in collaboration with IT and database managers. Both tasks were performed in parallel and in a synergistic way to consistently support the entire data management lifecycle for data analysis, and they are ongoing for additional data types. While the BEAT-COVID project group was maintaining one-hour bi-weekly video calls for general update and logistic discussions, specific video calls were set up with the required experts and duration for the topic at hand. These regular and iterative meetings with all stakeholders were necessary to enable the development of optimal semantic modelling and computational standardization.

### Representing patient data as FAIR digital objects

Central to our approach to implementing FAIR principles ‘for machines’ is the composition of ontological models from existing commonly used ontologies. These models serve as reference for the data in the source systems, creating a larger ‘virtual’ data warehouse. In this section we present the ontological models and FAIR infrastructure that were set up to represent patient data as FDOs discoverable for analytics. FDOs are broadly speaking a digital object identified by a Globally Unique, Persistent and Resolvable IDentifier (GUPRID) and described by metadata [6]. In the Materials and methods section, we explicitly describe how we represent patient data as FDOs where GUPRIDs role are defined.

#### Ontological data model for interoperability of clinical measurements

**Fig. 2:**
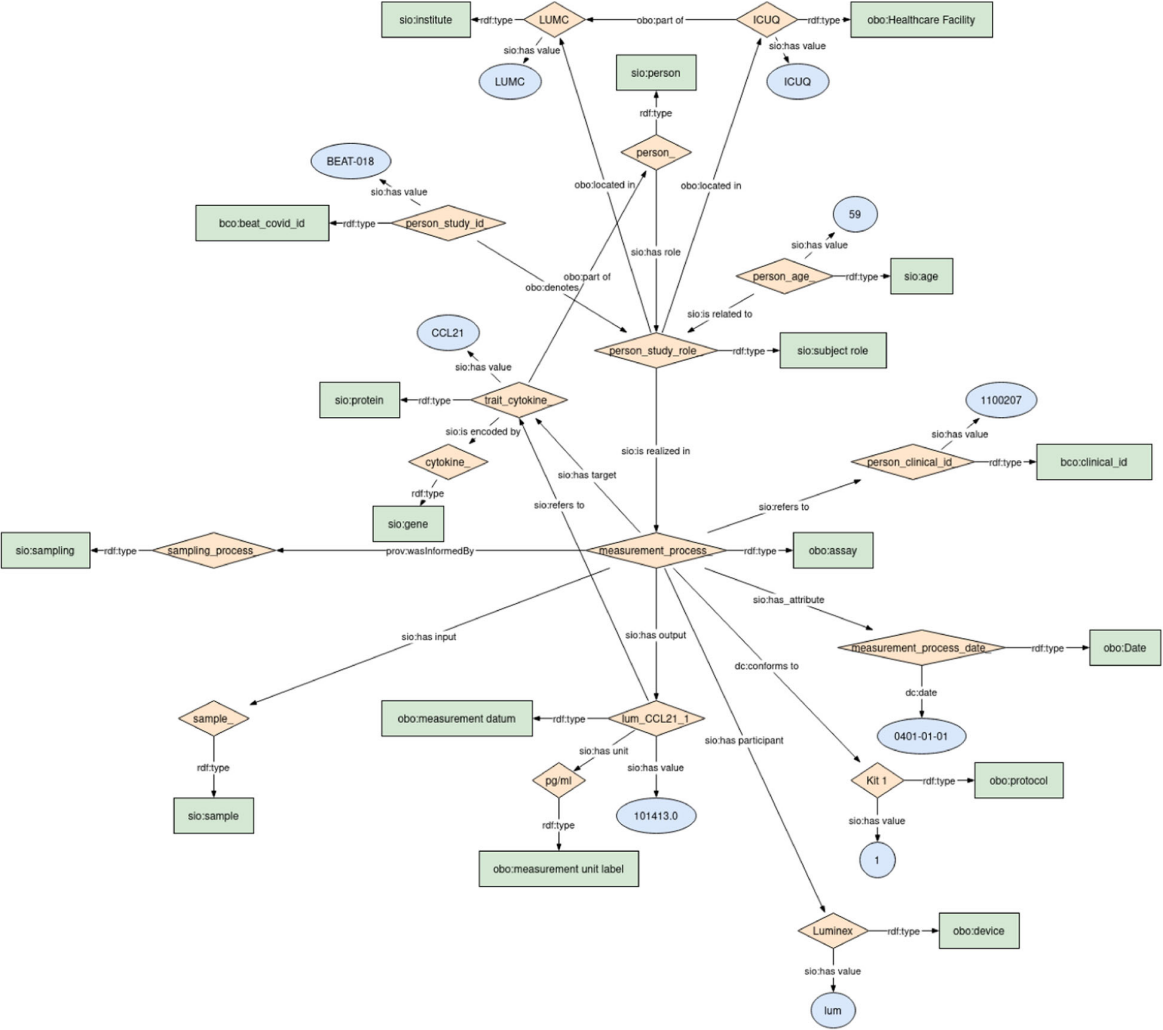
Ontological data model for the cytokine measurements patient dataset

To create a user centred research driven data infrastructure, we used the medical research questions as drivers for the data modelling. Important for our approach was to enable a high level of interoperability of patient data within the hospital. To that end we targeted all the FAIR principles that enable interoperability, which are I1 ((meta)data use a formal, accessible, shared, and broadly applicable language for knowledge representation), I2 ((meta)data use vocabularies that follow FAIR principles), and I3 ((meta)data include qualified references to other (meta)data). We first created a general concept model for the questions to extend with relevant clinical data, and mapped recurrent important terms mentioned by medical doctors into terms in Open Biological Biomedical Ontologies (OBO) ontologies [30, 31] described in the Web Ontology Language (OWL) [32]. When we received the first actual data, cytokine measurements on samples collected from clinically admitted patients, we created an ontological model in Resource Description Framework (RDF) [33] for this data (see Fig. 2). The cytokine model is based on the core semantic model that was developed in the EJP RD for common data elements in rare disease patient registries. This is a simple model that abstracts that every element in a patient registry is the outcome of a process, so that **process** becomes the core concept of the model [34]. We reused this model jointly with the quantitative trait semantic model [35] to capture clinical data measurements, where the ‘process of measurement’ is the core concept. Reusing these existing ontological models for observational data in the LUMC supports FAIR data. Not only does it allow interoperability with patient registries and quantitative traits, but also the common biomedical ontologies used allow data integration with external knowledge such as LOD.

**Fig. 3:**
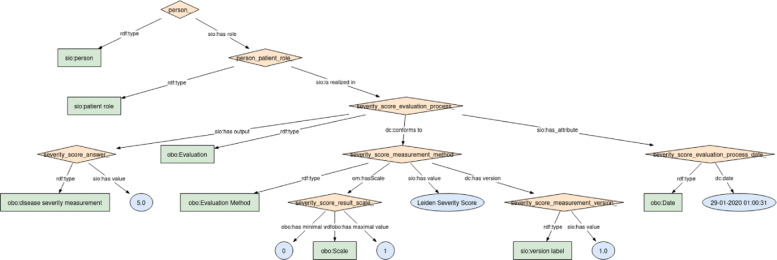
Semantic module to represent disease severity score phenotypes calculated in the hospital

We also modelled a new semantic module for disease severity score phenotypes following the same EJP RD core model, see Fig. 3. Apart from tracking the Apache IV Severity Score [36] and the SOFA Severity Score [37], medical doctors defined the Leiden Severity Score to obtain daily scores of disease severity for both COVID-19 patients admitted to the ward and ICU (Intensive Care Unit), more detailed information in the Materials and methods section. All these scores are based on lab results and clinical data and reflect the actual disease severity of the patient on that day and are informative for doctors to make decisions about patient care management. The ontological linking data model, and its modules (lab measurements, biosamples and disease severity score), are publicly available on GitHub-datamodel .

#### Ontological metadata model for COVID-19 resources

**Fig. 4:**
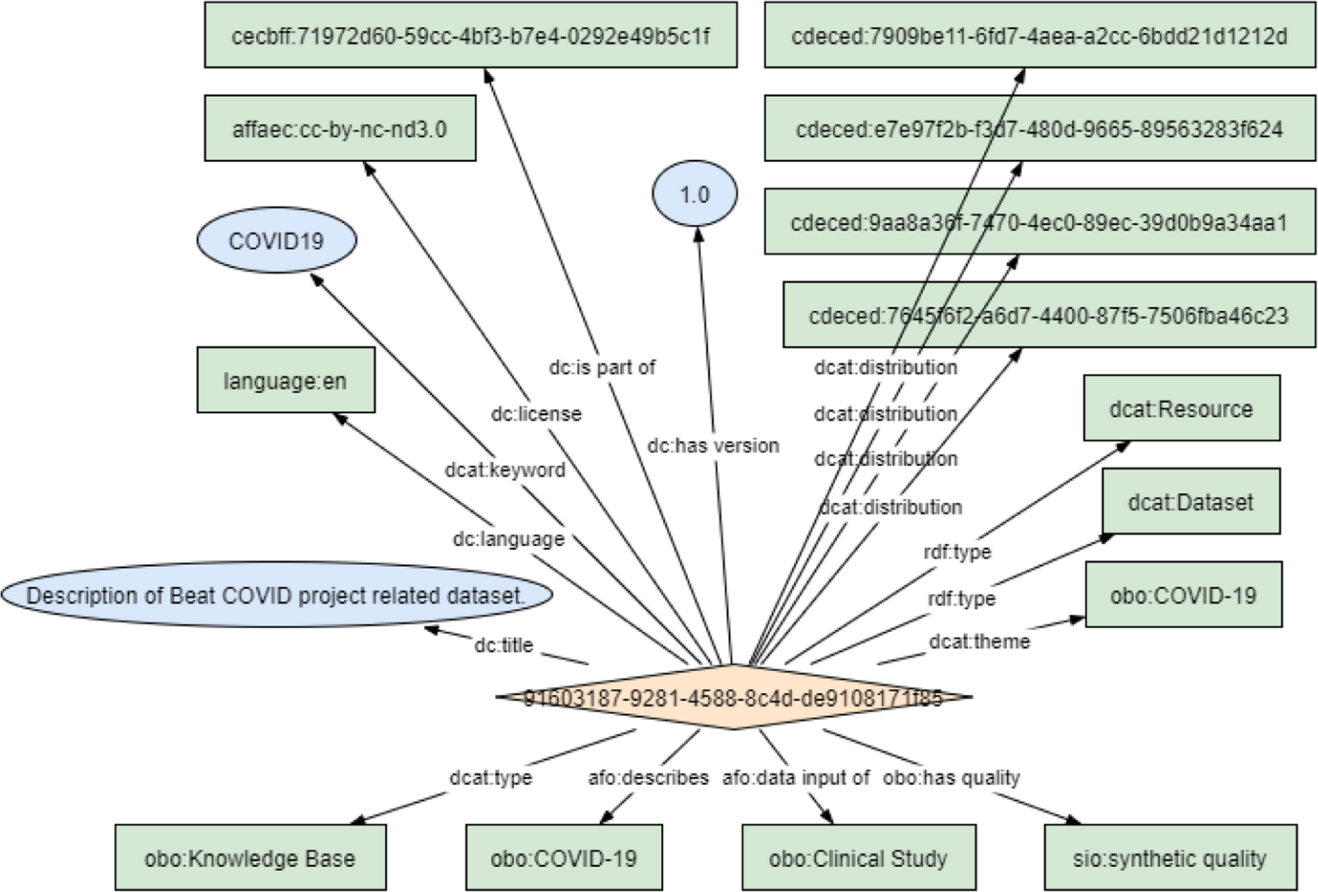
Ontological metadata model instantiated as an RDF graph. The four lower edges are the four additional metadata elements for COVID-19 data resource description

To allow the metadata of COVID-19 resources in the hospital to be findable, accessible, and reusable by both humans and machines, we provided an ontological model to expose it in a machine readable way. The FAIR principles that we prioritised were those about the use of globally unique and persistent identifiers for data and metadata, and providing rich metadata. We also followed the best practice of using *resolvable* identifiers. In particular, for findability we targeted F1 ((meta)data are assigned a globally unique and persistent identifier), F2 (data are described with rich metadata), and F3 (metadata clearly and explicitly include the identifier of the data it describes), for accessibility A1 ((meta)data are retrievable by their identifier using a standardized communications protocol), and A1.1 (the protocol is open, free, and universally implementable), and for reusability R1 (meta(data) are richly described with a plurality of accurate and relevant attributes), R1.1 ((meta)data are released with a clear and accessible data usage license), R1.2 ((meta)data are associated with detailed provenance), and R1.3 ((meta)data meet domain-relevant community standards). We targeted these principles to enable a high level of machine actionability for evidence-based analysis within the hospital and across public biomedical research resources. Not yet prioritised were F4 ((meta)data are registered or indexed in a searchable resource), A1.2 (the protocol allows for an authentication and authorization procedure, where necessary), and A2 (metadata are accessible, even when the data are no longer available), because federated discovery and learning with real world observations *across* hospitals is planned for future iterations of FAIRification. A1.2 is especially relevant in the case of sensitive patient health data. In practice, we designed a model by extending the DCAT2 based metadata model^1^ that is to manage the metadata of common datasets. With four additional metadata elements from three standard ontologies, including the property “TYPE” from the DCAT2, the properties “DESCRIBES” and “DATA INPUT OF” from the Allotrope Foundation Ontologies (AFO)^2^, and the property “HAS QUALITY” from the OBO Relations Ontology (RO)^3^, the metadata model features finer semantic granularity. In Fig. 4, we show how we can specify that the BEAT-COVID data resource in our project is a *knowledge base*, that describes *COVID-19*, that is supposed to contain data input of *clinical studies*, and that has *synthetic quality* by means of these four object property values or edges in the RDF graph. This makes the structured semantics of the metadata of COVID-19 resources richer and more precise. The metadata model is publicly available on GitHub-metadatamodel .

#### FAIR data point for assessing the metadata of BEAT-COVID patient data

**Fig. 5:**
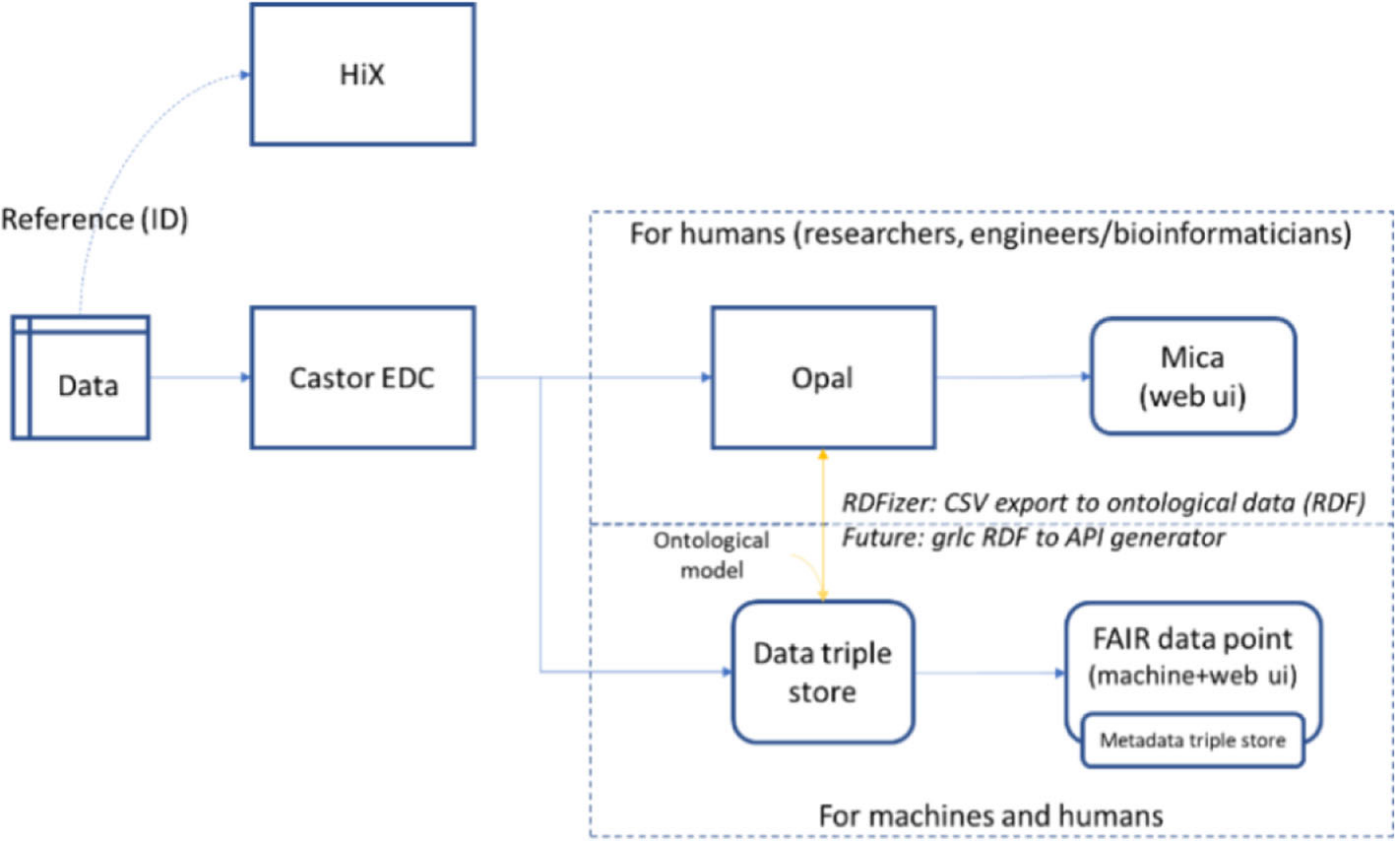
Integration of our ontological approach with existing systems

The basic idea of an FDP is to support scalable and transparent “routing” of data resources through stored metadata. The metadata stored and managed by an FDP makes the data resources described by the metadata semantically findable and reusable by machines. As an open gateway, it also makes different data resources accessible under defined constraints. Based on the designed ontological metadata model, we implemented an FDP to describe datasets in Opal and to publish FAIR metadata of these datasets on the Internet as complementary to the Mica system. This FDP publishes structured metadata for machines to automatically find BEAT-COVID datasets and to interpret how to access and use the data stored in Opal, for instance to those algorithms visiting the data with the right access (Fig. 5). Important to the FDP approach is that the data never leave its repository thereby protecting patient data and ensuring only authorized users have access. We performed an automated FAIR assessment of the same dataset from Mica described in the FDP. The results can be found here and showed that various aspects of the metadata description were improved in comparison to the Mica analysis results. For instance, FDP evaluation resulted in better identifier description of the (meta)data. With the publication of the BEAT-COVID resource metadata into the FDP we expect to increase the discoverability of COVID-19 patient data in the LUMC and to enable federated analytics for extended populations. To point out that an FDP is accessible and readable by machines through a REST API, and by humans through a Graphical User Interface (GUI). Note that the BEAT-COVID resource metadata is not all human readable. This is because the GUI of the current version of FDP only renders to the last fragment of a URI (Uniform Resource Identifier). For instance, the URI “www.example.org/ExOn/description” renders to the label “description” and the URI “www.example.org/ExOn/EL_00001” renders to the label “EL_00001”. We are working on a more appropriate solution to display the “LABEL” property from RDF Schema^4^, following the best practice to always provide this label for humans. The FDP is publicly available at https://w3id.org/biosemantics-lumc/beat-covid/fdp/.

#### Integrating the ontological models with the existing research data warehouse

Our next step was to add access to patient measurements as instances of the ontological model (‘ontologised data’) as a feature to the existing RDM. In Fig. 5, we show how ontologised data is integrated with the existing Opal and Mica data management system. Our objective was to use the Opal and Mica systems as a foundation for FAIRification in the LUMC. While the Opal system manages integration of datasets in the hospital, the Mica system adds valuable metadata about the data resources. Even though Opal and Mica do not directly provide semantic modelling functionality, they do provide a basic annotation functionality that we used as the basis for connecting the ontological models. To instantiate the ontological linking data model in RDF, we developed an ‘RDFizer’ Python script as a minimal prototype for patient data FAIRification (see yellow arrow from Opal to Triple Store in Fig. 5). Our current prototype uses CSV files with synthetic cytokine data as *input* to connect data from Opal to the ontological model that we developed for this data, thereby creating ‘ontologised data’ in RDF. Opal allows exporting datasets to CSV through its export function API^5^.

Conversely, REST Web APIs can be generated from the ontologised data using the grlc server [38] (see yellow arrow from Triple Store to Opal in Fig. 5). grlc is a tool to automatically convert SPARQL queries into REST Web APIs and make selected RDF data accessible to the Web. Moreover, it can translate SPARQL [39] queries stored and documented in GitHub repositories to Linked Data APIs on the fly. Essentially, it includes an additional DCAT2 data distribution interface (REST APIs) on top of the existing SPARQL endpoint. To demonstrate this additional way of reusing FAIR patient data, we implemented a set of Web API endpoints to retrieve patient data in RDF. We first developed data retrieval SPARQL queries, and then we ‘decorated’ and uploaded them in a GitHubrepository-grlcqueries to be interpreted by the grlc server and build the REST API interface automatically. The SPARQL queries are examples of the potential power to execute sophisticated federated analysis that can be extended as more data resources become available. The Web API endpoints are publicly available at http://grlc.io/api-git/LUMC-BioSemantics/beat-covid-RESTful-API.

### Querying FAIR patient data with LOD for medical questions

To showcase that the FAIR RDM and the derived data infrastructure allow answering medical questions by querying patient data in terms of the ontological model and together with external open science knowledge, we performed two simple SPARQL queries on the synthetic cytokine data (Table 2). The queries were defined to answer the initial real world medical doctors’ hypothesis related to cytokines FAIR data. From clinical practice, doctors observed different disease courses with different cytokine related immune responses and different prognoses, and potentially different disease molecular mechanisms. To personalize different treatment strategies, doctors need to know what the clinical parameters are that can be used as biomarkers for predicting the disease course of a patient. Cytokine levels could be such biomarkers. To stratify patients, we first defined the query to count the number of patients in the LUMC. Then, we defined a second query to link each measured clinical parameter with biological protein information from external sources in order to build patient cytokine profiles that can characterize individual immune responses at different time points. Queries such as these provide the basis for further analysis of prognostic indicators and disease mechanisms.

**Table 2:** Example queries using external LOD resources

Question	Result
Count number of patients	LUMCquery
Retrieve measured cytokines in the LUMC with protein annotation from the UniProt knowledgebase	Federatedquery

**Fig. 6:**
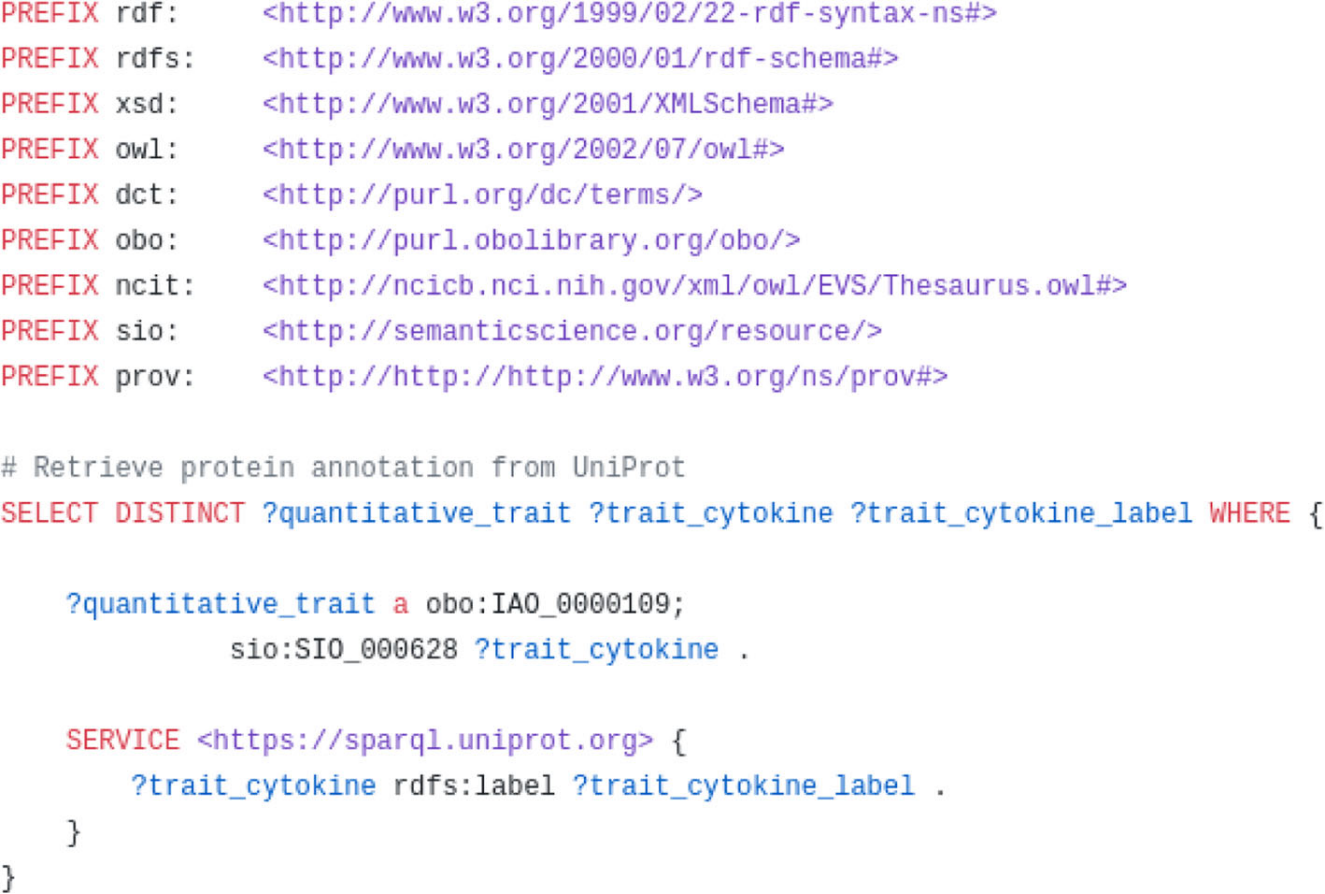
Federated SPARQL query crossing FAIR patient data with the UniProt knowledgebase

The first query demonstrates that clinical information from the LUMC can be queried, while the second demonstrates that queries can run across LUMC clinical data and external biomedical databases such as the UniProt protein knowledgebase by means of the federated SPARQL query shown in Fig. 6. The SPARQL queries are available on GitHub-queries . The aforementioned grlc server provides an additional REST Web API for these queries.

## Discussion

### FAIRification in the hospital

The COVID-19 pandemic revealed how critically important it can be that patient data from multiple systems in the hospital are prepared for instant integrative analysis across those systems, as well as across hospitals and countries. This would be feasible if the hospital had a FAIR RDM plan that implied making patient data available as FDOs and thereby findable, accessible, interoperable, and reusable for computers [1]. However, COVID-19 patient data are not yet natively collected as FAIR data. Therefore, we have described a strategy to facilitate the adoption of the FAIR principles in the hospital based on the FAIR architecture shown in Fig. 1 that complements an existing data management infrastructure. The strategy applies ontologies to increase the interoperability and machine readability of patient data records and patient datasets. We demonstrated that in the hospital (i) ontological models can complement existing data infrastructure, and (ii) they are an appropriate mechanism to formally capture agreement between stakeholders on what their data mean. They combine precise semantics for humans and corresponding actionable semantics for computers. Additional benefits are that they are extendible and they allow replacement with an improved ontological model (or adding multiple models). A similar ontology based approach is also applied to provide patient derived data as FDOs in biomedical and rare disease research such as in the EJP RD [21]. Interestingly, the results that we reused from the EJP RD project were addressing similar requirements as we had for COVID-19 data.

### Coordination with different stakeholders

The development of the FAIR RDM plan was made possible by a coordinated interdisciplinary effort. In our experience, FAIRification requires at least data producers, data consumers, and FAIR data modellers [13, 40]. This is because the essential step of capturing the meaning of data in terms of ontologies requires the combined expertise of these stakeholders. In our case, this was available through the BEAT-COVID collaboration. The collaboration is providing user needs, technical requirements, insight in existing procedures and best practices regarding the management of the data lifecycle in the hospital. A clear challenge for our FAIRification process was communication between the different stakeholders with very different backgrounds. This was further hampered by the communication limitations due to the pandemic itself. To mitigate the communication gap, we recorded meetings and shared material that was presented during the meetings. We also plan to organize Bring Your Own Data workshops to make stakeholders who are not FAIR experts more aware of the advantages that FAIR brings [41–43]. Under pressure of the urgency of the pandemic, we worked without dedicated FAIR stewards for this project. However, in going forward, this role seems essential to manage the necessary communication between disciplines [44].

### Establishing goals for FAIRification

Questions of researchers in the hospital were used as the drivers to establish FAIRification goals and to plan a FAIR RDM. The FAIRification preparation consisted of several meetings with medical doctors and clinical researchers. The focus of the meetings with domain experts was two-fold: (i) to identify the FAIRification goals, and (ii) to extract a set of specific research questions that drive the (meta)data modelling step. Both aims are related, because being able to answer *at least* the driving research questions is one of the main goals of FAIRification. The list of research questions included ‘What are the clinical parameters that can predict the disease course of a patient?’, ‘What are the biological pathways underlying patient symptoms and disease phenotypes?’, ‘How could biological pathways be positively or adversely affected by a particular treatment?’. The results of these meetings were guiding how data in the hospital should be interrelated and in what context they should be interpreted. We used this to define domain semantics in the context of testing and generating hypothesis with the help of OWL ontologies. The extendibility of ontologies mitigates the risk of limiting applications, because of initial overfitting on driving questions. Wider reusability of the FAIR RDM is a primary objective. To ensure that we are correctly capturing the semantics of knowledge and data, we are also exploring a formal method to validate the (meta)data models by the use of Competency Questions (CQs) and goal modelling. This will again rely on working with domain experts in close interdisciplinary collaboration. These research questions also facilitate communication between people of different expertise.

### Technical and social challenges and opportunities

For developing our approach within the BEAT-COVID collaboration, we took into account (i) the emergency of the situation, (ii) that various data management systems are in place at the hospital, (iii) that different types of data need to be prepared for timely exchange and efficient research. Consequently, our challenge was two-fold (i) to adapt our generic FAIRification workflow [12] in a hospital setting, (ii) to require minimal technical knowledge transfer, taking the opportunity of the combined expertise in the hospital that BEAT-COVID brought together. Key to our method is the development of two ontological models, one to enable analysis across clinical data (e.g. symptoms), investigational parameters (e.g. cytokine measurements), and data outside of the hospital, and another to represent the metadata of the patient data resources to increase the findability, accessibility and reusability. A metadata store was deployed conform to the FDP specification to provide access to this metadata. The metadata also includes a reference to access the ontological data. We demonstrated that Linked Data and Semantic Web technologies such as OWL ontologies, Triple Stores and the SPARQL query language provide the means to query patient data across sources in terms of the ontologies (Table 2). Taken together, these provide the FDOs for COVID-19 patient data and the basis for instant integrative federated analysis in the hospital.

While our ontological models aim to reflect our shared understanding of the data, a lack of tools still makes it challenging to transform health data to common data models such as HL7 FHIR [45], and for publishing it to findable resources [46]. There is a need for FAIRifier tools that support stakeholders in a clinical setting in every step from FAIR RDM planning to FAIR data creation, publication, evaluation, and reuse. Integration of FAIR implementations in existing data management tools such as Castor EDC can lower the burden substantially [40]. Similarly, the vocabulary and annotation features of Opal and Mica provide handles for future integration of FAIRification. The reuse of an abstract ontological data model, such as the EJP RD core model, in combination with the implementation of FDPs may further reduce thresholds for implementation and FAIR data reuse. An additional practical and technical challenge thereby is to protect patient identifying information but at the same time to have clinical data available close to real time. Classically most studies would retrieve data in retrospect from patient records. However, in the combat against COVID-19, first analyses were done when patients where still hospital admitted. Advanced data encryption was used to retrieve daily updates from patient records without including retrievable patient identifiers in the research data infrastructure. Although the big commitment of the BEAT-COVID group is facilitating the progress, other challenges for FAIRification in the hospital were ’social’, presumably because stakeholders are not familiar with the steps that are needed to make a resource reusable by computers across multiple locations. We propose that a FAIR data policy is put in place for health research data conform [47]. To pave the way, there are several ongoing efforts to meet the need for education, such as FAIR training for researchers, clinicians and different types of stakeholders in organizations such as ELIXIR TeSS [48] and the EJP RD project for rare diseases.

### Patient data accessibility hurdles

Protecting patient data and privacy is a major concern and it is part of FAIRification to make a clear reference to how data are protected. As researchers, we must establish data management mechanisms that ensure that patient privacy is preserved and its usage under control. There are several options to deal with data privacy and safety such as using anonymised datasets, using substitute synthetic representations of sensitive datasets, and having the legal and ethical framework in place for the processing of sensitive personal data in the sense of the GDPR. As first step, the hospital needs to develop and implement a data governance policy that clearly specifies how to extract and apply the data as approved by the patient in the informed consent. Delaying data governance may delay the FAIRification process because it needs to be clear what data will be available and in which form to plan the FAIRification, but also to specify data governance in the metadata of the resource when an algorithm visits the data to use. Then, underdeveloped metadata in data accessibility and data privacy hampers interoperability outside of the hospital. Consequently, it hampers data visiting, which means it hampers federated query and learning over FDPs and, therefore, limits hospital research capacity for analysis. Also, very important for accessibility and data privacy is that the digital objects *per se* can accommodate the criteria and protocols necessary to comply with regulatory and governance frameworks. Ontologies can aid in opening and protecting patient data by exposing logical definitions of data use conditions. Indeed, there are ontologies to define access and reuse conditions for patient data such as the Informed Consent Ontology (ICO) [24], the Global Alliance for Genomics and Health Data Use Ontology (DUO) standard [25], and the Open Digital Rights Language (ODRL) vocabulary recommended by W3C^6^. The first two are OBO ontologies for the formal specification the former of the patient informed consent and its process for research studies in the medical field, and the latter of the consented data use conditions and restrictions for research with large genomics and health data repositories. We are furthermore considering if the ODRL can serve as a common language to express access permissions for machines, similar to how DCAT2 provides a common language for resource metadata. Finally, it is worth noting that privacy preserving methods are available if data of the same person in multiple systems are required for a federated analysis [49, 50].

### International adoption of the FAIR principles for health data of hospitalised patients

The method for FAIRification that we described is focused on patient derived health data, down to the data record level. Two main outcomes are that we produced FAIR data for hospitalized patients, and we demonstrated that this data is instantly reusable for various secondary uses: for building software applications (and analysis workflows) via REST Web APIs, for querying cross-domain patient data and open public knowledge to add richer context to answer healthcare questions. While there are several projects that develop FAIRification procedures, they predominantly focus on life sciences data [14, 15, 51]. FAIR data in health is gaining momentum, and we already can find dedicated projects such as FAIR4Health [52] to use FAIR data in health to improve research. Our method has the same basis as the procedure followed earlier for rare disease patient registries (e.g. VASCA [13]), but here we integrated it with the hospital infrastructure, and demonstrated how the adoption of FAIR principles can be facilitated in the hospital through interdisciplinary collaboration. Hence, our experience may be valuable to national and global consensus on implementing FAIR principles in hospitals by the clinical community. For instance, the Dutch national Health Research Infrastructure (Health-RI) has stated that data stewardship at the Dutch University Medical Centres should adhere to the FAIR Principles [53]. Similar nationwide initiatives to improve health data reuse can be seen in Switzerland (Swiss Personalized Health Network [18]) and Germany (NFDI4Health [19]). These initiatives rely on a federated infrastructure, enhanced data interoperability and data linkage in compliance with privacy regulations for research. Our example has shown that FAIRification within the hospital can contribute to this infrastructure.

### Limitations and future work

We observed a number of limitations of our approach to enabling instant analysis of COVID-19 data across multiple hospital systems. First, we observed that the interdisciplinary collaboration and the willingness to implement FAIR principles, because of the pandemic, are not sufficient to provide easy access to data for implementing the FAIR services. A partial solution, at least to speed up the deployment of the FAIR services, could be to have synthetic patient data available. This could, for instance, be instantiated by Synthea [54, 55] from data in HL7 FHIR format. Second, at this time we have not incorporated a way to formally express patient consent and data usage conditions in our FAIR metadata. Currently, there are several efforts in human data communities to identify which elements are required, and standards are under development to capture these in machine readable ontological form, such as by ICO and DUO. These can be linked into our FDP metadata model in the future. Third, we have not specifically addressed tooling (including standards) to support hospital data stewards in FAIR data management. This could pertain to tools for capturing FAIRification goals, ontological data modelling, data conversion, and mapping. Data modelling and mapping were the most time consuming steps. For some of our data types it was difficult to identify an appropriate ontology term that we could incorporate trivially in our OBO-based application ontology. For instance, to map 103 cytokine measurement *datum* types, we needed two different ontologies^7^,^8^), which is not a best practice. The majority could be mapped to the Experimental Factor Ontology (EFO) [56], which is not an OBO ontology. And, we could not find some specific data types in any ontology. Therefore, we mapped them to a more general class, for instance we mapped specific interleukins measurement *datum* types such as for ‘interleukin-11’, ‘interleukin-26’ or ‘interleukin-32’ (among others) to the general data item class ‘blood interleukin measurement’^9^, which is the superclass of ‘blood interleukin-6 level’ class^10^, or we mapped specific measurement process types such as for ‘Tumor Necrosis Factor Ligand Superfamily Member 14’ cytokine to the general process class ‘Cytokine Measurement’^11^. We expect new limitations once we analyze new omics datasets and clinical observations. Also, tools that evaluate the ‘FAIRness’ of data can guide the FAIRification process. This partly depends on the standards used by the domain of the data community providers [57], but it is not always clear what these standards are, if any. Current ongoing work in the FAIRification ’world’ is to identify these community specific FAIR requirements and implementation choices. For instance, we envision as future work the establishment of FAIR maturity indicators for clinical data. Finally, we aim to progress on the opportunities for advancing research with FAIR patient data, further developing a FAIR Web API service to complement Opal APIs and knowledge graph based learning techniques. We would like to highlight the following developments.

#### Evaluation of ontological data models

We are evaluating the ontological models using CQs that are based on realistic questions posed by data model users [58], which are proposed as means to verify the scope (e.g., what is relevant to solve the challenges) and the relationships between concepts (e.g., check for missing or redundant relationships). A preliminary set of CQs from meetings with domain experts is available on GitHub-CQs .

#### COVID-19 hypothesis generation tool

We are developing a COVID-19 Hypothesis Generation tool for the LUMC based on the structured reviews for data and knowledge driven framework [59], as a means to exploit the FAIRification work for aiding medical doctors and researchers to answer their research questions. This framework has previously been used to support rare disease researchers to explore hypotheses as paths in case specific knowledge graph for their observations in the lab. After creating a preliminary knowledge graph with the FAIR synthetic cytokine data, we aim to incorporate background knowledge. The preliminary knowledge graph is available for browsing at LUMCBEAT-COVIDKnowledgeGraph .

#### Federated analytics across hospitals

We also aim to show how this FAIR infrastructure allows to query FAIR data from the BEAT-COVID project in the LUMC across other hospitals’ FAIR data without data leaving their source, i.e. the ‘data visiting’ approach. In the VODAN project, the GO FAIR VODAN in a box FDP [60] was used to test the trains and tracks of the PHT concept [61] and demonstrated the first intercontinental FDP SPARQL VODAN Africa proof of concept [62] developed by VODAN Africa and Asia - GO FAIR [2] query AllegroGraph WebView [63]. Secure FDP technology testing must be developed to implement trusted access control policies and to enable visiting synthetic datasets and pseudo-anonymised healthcare data. We aim to build on the VODAN and TWOC experiences and prepare an FDP instance that publishes BEAT-COVID metadata to be automatically found and used in trusted automated analytics workflows across multiple hospitals.

## Conclusion

We demonstrated that a FAIR research data management plan approach based on ontological models, open Science, Semantic Web technologies, and FDPs is a powerful method for generating FAIR patient data *at source*. FAIRification is providing data infrastructure that improves findability, accessibility, interoperability and reusability of patient real world observations in the hospital. Most importantly, we shown that FAIR patient data is machine actionable as digital objects linkable to LOD for analysis and ready to be used to develop applications for hypothesis generation and knowledge discovery on top. Finally, this work (in progress) showed what FAIRification entails in a real world hospital situation with existing infrastructure, different stakeholders and departments and the GDPR, and we discussed obstacles, challenges, solutions and future directions. We aim to provide a state of the art research data infrastructure in the hospital to deliver a federated solution enabling data access across the country and international borders, and accelerating research and translation to healthcare.

## Materials and methods

### Materials

#### FAIR digital objects and globally unique persistent identifiers (GUPRIDs)

The FAIR principles, specifically F1, include the requirement that metadata and data should be identified by GUPRIDs. In addition to this, the FAIR principle A1 requires that metadata and data are retrievable by their identifiers using a standardized communications protocol. As such, we set up our persistent identifiers according to these requirements for data and metadata (and the FDP itself as well):

**Data** The patient synthetic cytokines lab measurements dataset, which in turn is described by metadata records as FDOs themselves, is identified and retrievable by the *W3ID* persistent identifier service^12^ base https://w3id.org/biosemantics-lumc/beat-covid/, e.g. the RDF distribution GUPRID is https://w3id.org/biosemantics-lumc/beat-covid/rdf/beat-covid.ttl, and accessible through the LUMCBEAT-COVIDFDP .

**Metadata** The metadata of the patient cytokines dataset is identified and retrievable by the *PURL* persistent identifier service^13^ base http://purl.org/biosemantics-lumc/test-fdp/dataset/ and the GUPRID is http://purl.org/biosemantics-lumc/test-fdp/dataset/91603187-9281-4588-8c4d-de9108171f85, and accessible through the LUMCBEAT-COVIDFDP .

#### Ontologies

We mapped to Open Biological Biomedical Ontologies or OBO ontologies to facilitate biomedical integrative analytics since these ontologies are developed to be interoperable, logically well-formed and scientifically accurate by the community following the OBO principles [30, 31]. For data annotation with OBO ontologies we mainly used the Ontobee software system^14^, the Ontology Lookup Service from the EBI^15^, and the NCBO BioPortal^16^ as search engines to find ontological terms. See the description of the ontologies used for each model below.

**Data model.** For basic knowledge representation in RDF: RDF vocabulary or RDF^17^, RDF Schema or RDFS^18^, DCMI Metadata Terms – Dublin Core or DCT^19^, XML Schema or XSD^20^. For general Science and provenance representation: Semanticscience Integrated Ontology or SIO^21^, The PROV ontology or PROV-O^22^. For biological and biomedical domain representation: OBO ontologies^23^ (such as NCIT, IAO, OBI, RO, CMO and LABO), the Experimental Factor Ontology or EFO^24^. For the BEAT-COVID study specific representation: The BEAT-COVID Ontology or BCO^25^ developed for the formal representation of cytokine data model in OWL2.

**Metadata model.** For basic DCAT based metadata representation: RDF Vocabulary or RDF^26^, Data Catalog Vocabulary - version 2 or DCAT2^27^, DCMI Metadata Terms – Dublin Core or DCT^28^, FOAF Vocabulary or FOAF^29^. For the BEAT-COVID study representation: RDF Schema or RDFS^30^, XML Schema or XSD^31^, FDP Ontology or FDP-O^32^, the W3C Linked Data Platform Vocabulary or LDP^33^, OBO ontologies^34^ (such as NCIT, MONDO, IAO, RO, OGMS, EXO and DO), Semanticscience Integrated Ontology or SIO^35^, Wikidata Vocabulary^36^, Allotrope Foundation Ontology or AFX^37^, the DataCite Ontology^38^.

#### Data

The BEAT-COVID dataset we based our ontological models was an anonymized longitudinal set of cytokine levels measured on COVID-19 hospitalized patients in the LUMC. We created a cut shorter and synthetic pilot version of the dataset to proof our concept approach for FAIRification in the hospital. We created the synthetic dataset using randomization functions in excel. The synthetic dataset contains 9 rows of measurement records on 103 cytokines performed in 4 different panels using Luminex technology. The dataset contains basic information for each record, such as the record timestamp, the date of sampling, the age of the patient, the date of measurement and the cytokine levels. Example to data records in tabular format is available on this GitHub-syntheticdatalink.

#### Software

We used several technologies in the different steps of our method. The FAIRification tools and versions used are described within each step in the Methods section below. The software and tools we used to build three different applications on top of FAIR data, were:

**Data analytics with Semantic Web technologies.** We used the W3C recommended SPARQL query language [39] to perform data analytics over the LUMC RDF patient data and across diverse external data sources in LOD. We used the free edition of GraphDB Triple Store v9.7.0, where the data is natively stored as RDF.

**Web API development.** We used grlc v1.3.6 [38] to enable programmatic access to FAIR data in the hospital. Grlc is a lightweight server that automatically builds consistent, well documented and neatly organized Linked Data APIs on the fly, with no input required from users beyond a URL path to a GitHub repository hosting a set of SPARQL queries that complies with the specific grlc syntax^39^. It provides three basic operations: 1. generates the Swagger spec of a specified GitHub repository; 2. generates the Swagger UI to provide an interactive user facing frontend of the API contents; and 3. translates SPARQL queries into HTTP requests to call the operations of the API against a SPARQL endpoint with parameters set in the queries.

**Hypothesis generation tool.** We used the Neo4j graph database framework [64] as used in the structured reviews approach [59] for storage, management and mining of FAIR patient data. The graph database technology has been shown to facilitate management and exploration of biomedical knowledge [65]. Neo4j graph database enables users to query the knowledge graph using the Cypher query language, either through an API or a GUI. RDF data was imported into the Neo4j Community Server v4.2.5 graph database through the Neo4j neosemantics toolkit v4.2.0 [66].

Note that we created a GUPRID for each software/service application based on the W3ID persistent identifier service, i.e. for the FDP, for the Triple Store, and for the Neo4j browser (see *Availability of data and materials* section).

### Methods

**Fig. 7:**
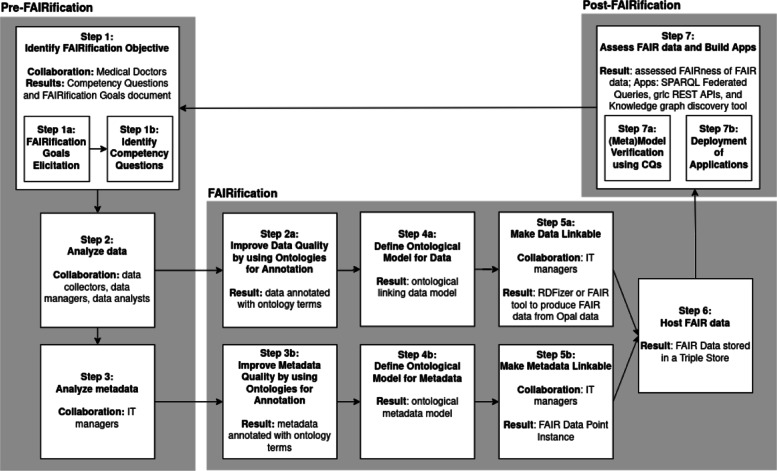
BEAT-COVID FAIRification workflow to make the data management and infrastructure in the hospital more FAIR. Collaborators and results are described in every step where applicable

We defined and implemented a method to make COVID-19 observational patient data in the hospital FAIR. This method is described in a detailed FAIRification workflow illustrated in Fig. 7 and is an adapted version of the workflow presented by Jacobsen et al. [12]. We explicitly add the result obtained in each step, where applicable. We also include in which steps the FAIR experts worked in collaboration with other members of the BEAT-COVID group.

### Pre-FAIRification

#### Step 1: identify FAIRification objective

The first step was to determine the objective for making COVID-19 observational patient data FAIR in the hospital to define the specific FAIR requirements, implementations and workflow of this study. Medical doctors have pressing questions at point of care such as ‘What are the clinical parameters that can predict the disease course of a patient?’, ‘What are the biological pathways underlying patient symptoms and disease phenotypes?’, and ‘How can a patient be positively or adversely affected by a particular treatment?’. The FAIRification objective was therefore to prepare the diverse COVID-19 observational patient data to answer these questions. To this end, data needs to be integrated in a network and systems medicine approach [67], combined with external biomedical knowledge, and ready for computational analysis as illustrated in Fig. 1.

#### Step 2 and step 3: analyze data and metadata

**Research data management in the hospital.** From admission date until discharge, patient data were collected by different departments. The types of COVID-19 observational data relevant for research, and so for FAIRification, were diverse: demographics information, clinical information, laboratory measurements, transcriptomics (RNA-Seq) data, metabolomics data, and if the patient was transferred to ICU, then data related to ICU outcome. The format depends on the different EDC systems used. Within LUMC, clinical and preclinical information were collected in HiX [68] and Castor EDCs [26], whereas ICU data was managed by the MetaVision software [69]. These EDC systems have different data access interfaces and use different technologies. To provide a single point of data access, research data were combined in the Opal data warehousing system. Opal is the OBiBa’s (Open Source Software for Epidemiology) core database application to store data in central data repositories that integrate under a uniform interface data collected from multiple sources, and it provides tools to import, transform and describe data [27]. Patient data was anonymised before importing it into Opal using advanced data encryption. Descriptions of the datasets, i.e metadata, stored in Opal were published on the Web through the Mica software application. Mica is used to create Web data portals for large scale studies or multiple study consortia. It provides a structured description of consortia, study catalogues and datasets, annotated and searchable data dictionaries, and data access request management. It is built upon a multitier architecture consisting of a REST application server for data management and administration, and clients to create and display data on the Web [28]. Opal and Mica are two standalone but interoperable software applications that provide features for management, harmonization, and analysis of epidemiological datasets [29, 70].

**FAIR analysis of COVID-19 observational patient data.** To improve the findability, accessibility, interoperability, and reusability of digital assets, we performed a FAIR analysis of (meta)data, i.e. an analysis of the FAIR status of data and metadata. We analysed data and databases to evaluate the FAIRification effort needed [12]. We started by analysing observational clinical measurements. We first got access to laboratory measurements of immunoresponse clinical parameters, cytokine levels, collected on different time points per patient to monitor its condition progress. Access to data was provided to us as an anonymised dataset. Then, we analysed the databases where these data were stored, which were first in Castor databases since this was the primary EDC system used in the hospital, second in Opal data warehouse since this system was used to integrate and store data from the various data sources. We investigated the representation (structure and format) and meaning (semantics) of the data, and the tools and technologies of each database system to optimize the FAIRification process of data.

### FAIRification

#### Step 2a and step 4a: improving interoperability with semantic web technologies and a linking data model

We described a synthetic cytokines dataset with ontologies. In Europe, GDPR imposes obligations onto organizations anywhere, so long as they target or collect data related to people in the EU. To comply with GDPR, we created a synthetic dataset of cytokine measurements, i.e. substituted synthetic representations of sensitive datasets, by using randomization for modelling patient data. This dataset contains basic information related to cytokine measurements and biosamples used per patient and time point, and a patient clinical identifier to link to clinical data. With the goals to answer research questions of medical doctors and make patient data machine readable to enable interoperability within data resources in the hospital and with external open science datasets such as LOD, we designed ontological models for cytokine lab measurements, biosamples and severity scores to represent data based on the Linked Data principles [71] and Semantic Web technologies such as the W3C recommended RDF and OWL standards [32, 33]. Our approach was to define a conceptual model as an abstract and reusable model to capture as much of patient data (measurements, biosamples and score phenotypes), by using standard common schemas and well established ontologies and vocabularies widely used by the biomedical community such as the ones in the OBO Foundry [30]. With this approach we created an ontological linking model for cytokines measurements dataset from the laboratory.

#### Step 3a and step 4b: improving findability, accessibility, interoperability and reusability with semantic web technologies, a metadata model and FAIR data points

With the goals to answer research questions of medical doctors and make resource metadata human and machine readable to enable cross-resource data analytics, we designed a metadata ontological model and implemented an FDP instance [23] to make LUMC COVID-19 digital objects findable for machines on the Internet. An FDP is a Web application that enables data owners to expose information about their datasets using rich machine actionable metadata. It allows creating, storing, and serving FAIR metadata about datasets and its distributions for both humans and machines. An FDP does *not* enable open access, but the metadata is expected to include information about what the resource contains and how datasets and content can be accessed under defined conditions. Opening up FAIR (meta)data by publishing them on an FDP allows algorithms to search these (meta)data, looking for patterns [72]. Mica is a tool to expose datasets from an Opal database on the Internet through Web portals that allow (meta)data descriptions. An FDP provides additional means to expose FAIR metadata, i.e. machine actionable, via the FDP specification, a standardized metadata ontological model based on DCAT [22]. FDP also exposes (meta)data via a REST Web API that enables client applications to automate retrieval, aggregation and filtering (meta)data from distributed FDPs. We used FDP v1.10.0.

#### Step 5 and step 6: make (meta)data as linked data and host FAIR data

To host and publish patient data, we cut the original synthetic cytokine patient dataset into a few rows. We generated patient Linked Data using this synthetic patient data we created as *input* and instantiating the linking data ontological model. To do it we developed ‘RDFizer’ a FAIRification tool in Python 3 that parses and converts the synthetic data CSV file into RDF. To host the generated FAIR data, we used the free edition of GraphDB Triple Store [73] v9.7.0 where the data is natively stored as RDF. We implemented an FDP instance where the metadata ontological model is described and published as DCAT based Linked Data.

### Post-FAIRification

#### Step 7: assessment and software applications

**Evaluation** We evaluated the discoverability of the BEAT-COVID resource by means of the FAIR Maturity Indicators evaluator tool [74]. We have evaluated our ontological models by means of several CQs [58] (in progress). We have answered the questions using SPARQL queries for the sake of reusability, then users can reuse the queries if they want updated answers in the future.

**Built applications on top of FAIR data.** We implemented three different applications: 1. SPARQL federated queries for data analytics with Semantic Web technologies; 2. Web API service for programmatic access; 3. Knowledge graph based hypothesis generation tool. See software details in the *Materials* section.

### Severity score calculation

The severity score is based on the 4C mortality score developed by Knight et al. [75]. The 4C mortality score is a prediction score calculated at admission. The severity score calculated in our cohort represents the daily clinical disease severity, and thus is dependent on parameters that can change from day to day. Therefore, the fixed parameters of the 4C score were removed (i.e. age, sex at birth, number of comorbidities), and daily oxygen flow for non-ICU patients (l/min) and p/f ratio (kPa) and FiO2 (%) for ICU patients were added to our severity score.

## Data Availability

The datasets supporting the conclusions of this article are available in the following repositories. The ontological models, the SPARQL queries, grlc SPARQL queries, SPARQL CQs and scripts are freely available at the Biosemantics (GitHub): The data model is available at https://github.com/LUMC-BioSemantics/beat-covid/tree/master/fair-data-model/cytokine/model-triples The metadata model is available at https://github.com/LUMC-BioSemantics/beat-covid/tree/master/fair-metadata-model Synthetic cytokine patient dataset in CSV is available at https://github.com/LUMC-BioSemantics/beat-covid/tree/master/fair-data-model/cytokine/synthetic-data Source code for RDFizer is available at https://github.com/LUMC-BioSemantics/beat-covid/tree/master/fair-data-model/scripts/rdfizer COVID-19 synthetic patient cytokine knowledge graph in RDF is available at https://github.com/LUMC-BioSemantics/beat-covid/tree/master/fair-data-model/cytokine/rdf RDF data is accessible through the LUMC BEAT-COVID FDP at https://w3id.org/biosemantics-lumc/beat-covid/fdp/ Source code for FDP implementation is freely available at the FAIRDataPoint at https://github.com/FAIRDataTeam/FAIRDataPoint RDF is queryable through the Beat-COVID Triple Store at https://w3id.org/biosemantics-lumc/beat-covid/triplestore/ SPARQL queries are available at https://github.com/LUMC-BioSemantics/beat-covid/tree/master/fair-data-model/cytokine/sparql-queries grlc endpoint APIs are available at http://grlc.io/api-git/LUMC-BioSemantics/beat-covid-RESTful-API grlc SPARQL queries are available at https://github.com/LUMC-BioSemantics/beat-covid-RESTful-API LUMC BEAT-COVID Knowledge graph is available for browsing at Evaluations: FAIR assessment results of a dataset described in Mica are available at https://fairsharing.github.io/FAIR-Evaluator-FrontEnd/#!/evaluations/4081, and the FAIR assessment results of the same dataset, but described in a FDP are available at https://fairsharing.github.io/FAIR-Evaluator-FrontEnd/#!/evaluations/5589 SPARQL CQs are available at https://github.com/LUMC-BioSemantics/beat-covid/tree/master/fair-data-model/cytokine/competency-questions Figures: All model figures both in this manuscript and in GitHub project repository were automatically produced using the corresponding RDF/Turtle file as *input* and the Web drawing tool at https://w3id.org/ejp-rd/tools/rdf-drawing

## Abbreviations

FAIR: Findable, Accessible, Interoperable and Reusable
VODAN: Virus Outbreak Data Network
TWOC: Trusted World of Corona
FDO: FAIR Digital Object
GDPR: General Data Protection Regulation
LUMC: Leiden University Medical Center
RDM: Research Data Management
EJP RD: European Joint Programme Rare Diseases
DCAT: Data Catalogue Vocabulary
FDP: FAIR Data Point
LOD: Linked Open Data
EDC: Electronic Data Capture
OBO: Open Biological Biomedical Ontologies
OWL: Web Ontology Language
RDF: Resource Description Framework
ICU: Intensive Care Unit
GUI: Graphical User Interface
URI: Uniform Resource Identifier
CQ: Competency Question

## Footnotes

1 https://www.w3.org/TR/vocab-dcat-2/

2 https://www.allotrope.org/ontologies

3 http://www.obofoundry.org/ontology/ro.html

4 https://www.w3.org/TR/rdf-schema/

5 https://opaldoc.obiba.org/en/latest/python-user-guide/export/export-csv.html

6 https://www.w3.org/TR/odrl-vocab/

7 The Clinical Measurement Ontology (CMO), OBO Foundry, http://www.ontobee.org/ontology/CMO

8 The Experimental Factor Ontology (EFO), EMBL-EBI, https://www.ebi.ac.uk/efo/

9 The ’blood interleukin measurement’ class from CMO, OBO Foundry, http://purl.obolibrary.org/obo/CMO_0001925

10 The ’blood interleukin-6 level’ class from CMO, OBO Foundry, http://purl.obolibrary.org/obo/CMO_0001926

11 The ’Cytokine Measurement’ class from the NCI Thesaurus OBO Edition (NCIT), OBO Foundry, http://purl.obolibrary.org/obo/NCIT_C74804

12 https://w3id.org/

13 http://www.purlz.org/

14 http://www.ontobee.org/

15 https://www.ebi.ac.uk/ols/index

16 https://bioportal.bioontology.org/

17 https://www.w3.org/2001/sw/RDFCore/Schema/200212bwm/

18 https://www.w3.org/TR/rdf-schema/

19 https://www.dublincore.org/specifications/dublin-core/dcmi-terms/

20 https://www.w3.org/TR/xmlschema11-1/

21 https://github.com/MaastrichtU-IDS/semanticscience

22 https://www.w3.org/TR/prov-o/

23 https://obofoundry.org/

24 https://www.ebi.ac.uk/efo/

25 https://github.com/LUMC-BioSemantics/beat-covid/blob/acd7aadef06643e160712eec5486b9e8ba484713/fair-data-model/cytokine/ owl/cytokine_ontological_model.owl

26 https://www.w3.org/TR/rdf11-concepts/

27 https://www.w3.org/TR/vocab-dcat-2/

28 https://www.dublincore.org/specifications/dublin-core/dcmi-terms/

29 http://xmlns.com/foaf/spec/

30 https://www.w3.org/TR/rdf-schema/

31 https://www.w3.org/TR/xmlschema11-1/

32 https://w3id.org/fdp/fdp-o#

33 https://www.w3.org/ns/ldp

34 https://obofoundry.org/

35 https://github.com/MaastrichtU-IDS/semanticscience

36 https://www.wikidata.org/

37 https://akswnc7.informatik.uni-leipzig.de/dstreitmatter/archivo/purl.allotrope.org/voc--afo--REC--2021--03--afo/2021.07.04-010558/ voc--afo--REC--2021--03--afo_type=pyLodeDoc.html

38 https://sparontologies.github.io/datacite/current/datacite.html

39 https://github.com/CLARIAH/grlc
